# Subclavian Artery Pseudoaneurysm Formation 3 Months after a Game of Rugby Union

**DOI:** 10.1155/2015/346192

**Published:** 2015-08-17

**Authors:** T. Evans, S. Roy, M. Rocker

**Affiliations:** ^1^Department of General Surgery, Royal Glamorgan Hospital, Llantrisant CF72 8XR, UK; ^2^Department of Orthopaedic Surgery, Royal Glamorgan Hospital, Llantrisant CF72 8XR, UK

## Abstract

Pseudoaneurysms of the subclavian artery remain a rare complication after fracture of the clavicle. We report a case of delayed diagnosis of a subclavian artery pseudoaneurysm after a closed fracture of the clavicle in a 15-year-old patient, 3 months after the original injury while playing rugby union. Despite several attendances to the Emergency Department with vague symptoms, the final diagnosis was confirmed by duplex ultrasound and Computed Tomography of the thorax. Surgical repair was indicated due to acute limb ischaemia from distal embolisation from a large pseudoaneurysm, with the patient making a full recovery. This case highlights the need for clinical vigilance when assessing patients, particularly on repeated occasions when their recovery appears to be impaired. A thorough history and clinical examination can raise suspicion of even rare occurrences and aid prompt management.

## 1. Case Report 

**Figure 1:**
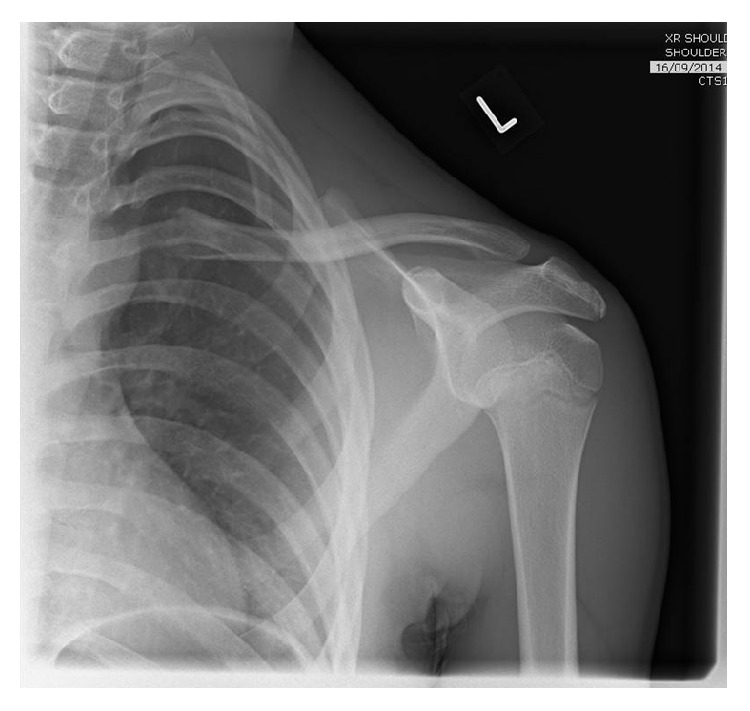
AP X-ray indicating minimally displaced mid-clavicle fracture.

A 15-year-old boy attended the Emergency Department (ED) with a short history of a pale left arm. He denied pain but complained of some altered sensation throughout his left hand. He had a significant recent medical history, having suffered a fractured midshaft of his left clavicle (see Figure 1) managed conservatively with a broad arm sling 3 months previously. Furthermore, he had 3 recent attendances to the ED with pain and “pins and needles” which felt like “a trapped nerve,” the first of which was a sudden onset of clavicular pain and altered sensation on throwing a ball. At each ED visit he was assessed, reassured, and discharged with documentation stating “no neurovascular deficit.”

Physical examination on his final visit revealed a warm but pale arm with no radial, ulnar, or brachial pulses palpable, but a normal capillary refill time of 2 seconds, and a reduced power in his intrinsic muscles of his left hand, but normal peripheral neurovascular examinations in remaining limbs and an electrocardiograph showing sinus rhythm. Chest X-ray confirmed no cervical ribs.

A duplex ultrasound scan showed a loss of flow in the brachial artery below the left elbow, with thrombus partially occluding the artery. Also noted was a 3 cm abnormality in the left subclavian artery that was thought to be a subclavian aneurysm that contained thrombus.

**Figure 2:**
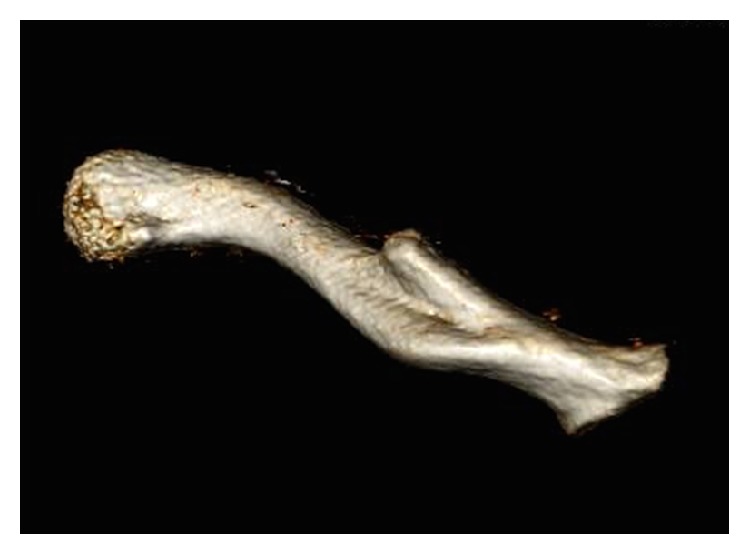
3D reconstruction showing posterior view of clavicle.

**Figure 3:**
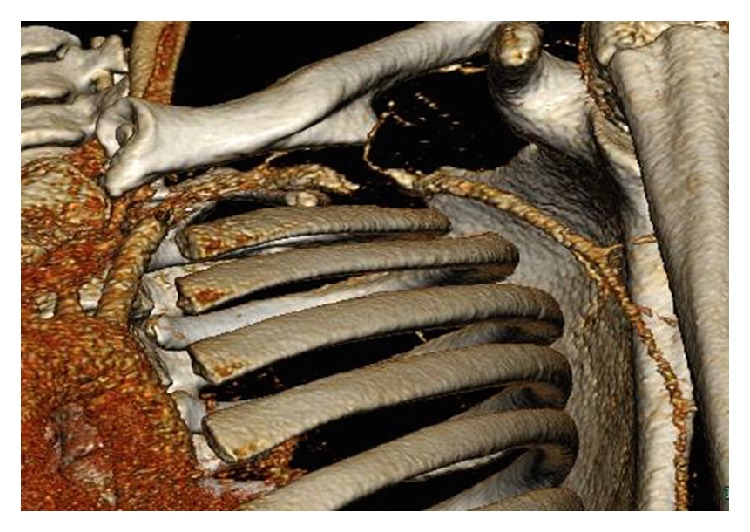
3D reconstruction showing filling defect and pseudoaneurysms in the left subclavian artery.

Subsequent Computed Tomography (CT) of the thorax (Figures 2 and 3) confirmed a diagnosis of a 4.5 × 3.5 × 2.5 cm false aneurysm of the subclavian artery lying on the superficial aspect of the anterior second rib and also showed evidence of the previous mid-clavicle fracture that had healed but malaligned. The CT also showed the distal end of the proximal shaft protruding posteriorly into the false aneurysm.

Transfer was arranged that day to the local acute vascular surgeon on call, where he underwent a subclavian-axillary bypass and brachial embolectomy. After 4 days of recovery as an inpatient including a heparin infusion he was discharged, symptom-free.

## 2. Discussion

The annual incidence rate of clavicular fractures is estimated to be between 30 and 60 cases per 100,000 people [1]. The most common complication following a clavicular fracture is nonunion, quoted as occurring between 6 and 15% of cases [2]. Neurovascular compromise is a rare but recognised complication of clavicle fractures. While case reports commenting on similar cases exist [3–5], they remain subtle to detect clinically. Kendall et al. reported a fatality from an isolated clavicle fracture from transection of the subclavian artery [6], and while this fatality was due to a large haemothorax and subclavian artery puncture from an unwitnessed fall, it shows the life-threatening potential of these fractures. Clavicular fractures were cited as the cause of 50% of traumatic subclavian artery injuries [1]. Arterial injury can result in puncture, pseudoaneurysm formation, or occlusion. Arterial occlusion leading to acute limb ischaemia is another severe complication. A subclavian artery pseudoaneurysm is therefore regarded as a potential risk to both the life and the limb of the patient and requires prompt management.

Arterial rupture usually causes life-threatening haemorrhages and must be carefully ruled out by physical examination as well as diagnostic imaging. Physical examination of the upper limb must focus on skin color, temperature, sensation, hand function, and presence of upper limb pulses. Contrast CT represents a key diagnostic exam, while angiography offers both a diagnostic and a therapeutic approach.

In 1983 Sturm and Cicero devised five criteria that lead the examining doctor to suspect an arterial injury [7]. These criteria include first rib fracture, diminished or absent radial pulses, palpable supraclavicular hematoma, chest X-ray demonstrating a widening of the mediastinum or haematoma over the area of the subclavian artery, and brachial plexus palsy.

Management options include both surgical and endovascular techniques. Surgical management remains a challenge and generally requires either a sternotomy or a supraclavicular and infraclavicular approach for safe proximal and distal control of the vessel. This approach risks significant blood loss and injuries to nearby neurovascular structures; nonsurgical options for management have been published and endovascular procedures including covered stents seem to reduce these risks but are often not available in the acute setting [8, 9]. They do, however, seem to offer a successful method of treating this difficult and rare condition, without the need for a major intrathoracic operation. Historically, endovascular repair reduced the risks associated with a traditional surgical repair requiring wide exposure to gain access to the injured vessels. The argument against endovascular repair was the clinicians inability to exclude other injuries associated with the vascular injury since avoiding surgery also means avoiding a chance to directly see such injuries at the time of operation. However, with the increasing accuracy and availability of radiology this may well be less relevant.

## 3. Conclusions

While subclavian artery pseudoaneurysm remains a rare complication after fracture of the clavicle, this case clearly describes a prolonged series of symptoms that show how important are a detailed history and physical examination along with an open mind that can help reduce risks to patients life and limb.
